# Advanced ANN Architecture for the CTU Partitioning in All Intra HEVC

**DOI:** 10.3390/s25195971

**Published:** 2025-09-26

**Authors:** Jakub Kwaśniak, Mateusz Majtka, Mateusz Lorkiewicz, Tomasz Grajek, Krzysztof Klimaszewski

**Affiliations:** Institute of Multimedia Telecommunications, Poznan University of Technology, pl. M. Skłodowskiej-Curie 5, 60-965 Poznań, Polandtomasz.grajek@put.poznan.pl (T.G.)

**Keywords:** artificial neural networks, HEVC, ResNet, DenseNet, video encoding

## Abstract

Due to the growing complexity of video encoders, the optimization of the parameters of the encoding process is becoming an important issue. In recent years, this has become an important field of application of neural networks. Artificial neural networks in video encoders are used to accelerate the video encoder operation. This paper demonstrates the use of different ResNet- and DenseNet-type architectures to accelerate the CTU partitioning algorithm in HEVC in All Intra mode. The paper demonstrates the results of an exhaustive evaluation of different proposed architectures, considering compression efficiency, network size, and encoding time reduction. Multiple pros and cons of the proposed architectures are presented in the Conclusions, considering various limitations that may be important for a given application, like hardware-constrained sensor networks or standalone small devices operating with images and videos.

## 1. Introduction

One of the most recent video coding technologies is High Efficiency Video Coding (HEVC) [1]. This video coding standard is used, among others, in digital TV broadcasting. HEVC offers improved coding performance compared to previous standards, especially for high-resolution videos. Its efficiency in terms of providing a high compression ratio and keeping the quality of the reconstructed images at acceptable levels made it possible to use also use HEVC in the compression of static images [2]. Although HEVC was standardized in 2013, the work on improving its operation is still ongoing. Especially in hardware-constrained or power-constrained applications, the older coding standards still find their use due to lower complexity and the remaining potential for optimizing their performance. Even the preceding standard, Advanced Video Coding (AVC), is still a subject of ongoing research [3]. The more recent Versatile Video Coding (VVC) standard is significantly more complex, and its adaptation to constrained environments is more problematic. The research on optimizing its performance is also ongoing, as evidenced by the recent papers [4,5].

In our work, we focus on HEVC mostly due to the fact that its intra-frame encoding quality is sufficient for encoding still images while being significantly less complex than VVC. Other researchers have also studied possible ways to improve the performance of HEVC. In [6], the authors focus on optimizing the rate control of HEVC, while our work focuses on a more time-consuming process of choosing the CTU divisions.

**Figure 1:**
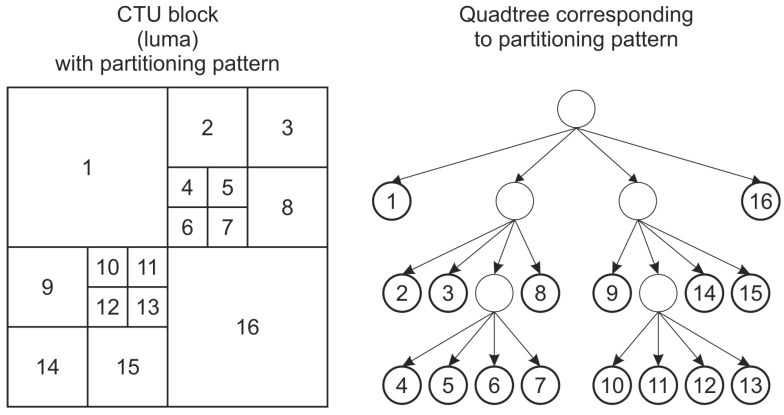
An example of a CTU division. The divided CTU is shown on the left and the corresponding quadtree on the right. Each leaf on the right can have its own division flag that signals whether it is further divided or not.

One of the properties of HEVC that makes it possible to obtain a high compression ratio is the use of advanced hierarchical division of the image into blocks. At first, the whole image or video frame is divided into blocks of the same size (usually 64 × 64 pixels) called a Coding Tree Unit (CTU). CTUs can be further divided using a quadtree structure into Coding Units (CUs) down to the size of 8 × 8 pixels. Those Coding Units undergo the coding process. The process of establishing the way the given CTU is divided is usually called partitioning. The complex structure of divisions makes it challenging to choose the optimal one. The division of CTUs of an image or video frame into CUs is the most time-consuming part of the encoding [7]. The example structure of divisions for a single CTU is presented in Figure 1.

**Figure 2:**
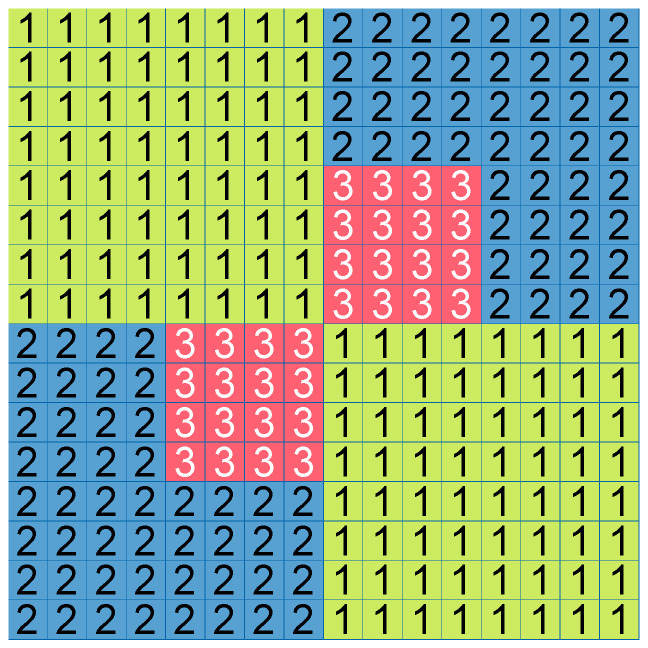
The division matrix for the same division as shown in Figure 1. The numbers indicate the level of the CTU division.

The division example shown in Figure 1 can be signaled as a set of division flags. In the implementation of encoders and decoders, the same information can be stored in a division matrix—a rectangular matrix of the size of 16 × 16 cells (for the CTU size of 64 × 64) that includes the information of the level of division for a given area of CTU. An example of such a matrix for the same case as shown in Figure 1 is presented in Figure 2.

The computational complexity of CTU processing is a widely studied problem among researchers. In most cases, the goal is to speed up the process while maintaining the compression ratio as high as possible. The baseline of this research is usually the reference implementation of an HEVC encoder, called then HEVC Test Model (HM) [8]. Different methods are employed to perform a fast and efficient partitioning of a CTU. One way to approach the problem of speeding up the partitioning process is to employ refined algorithms to perform partitioning based on different properties of the compressed image, such as the texture [9], the statistical properties [10], or the heuristic properties [11]. Those methods are usually used to recursively predict the division flags on consecutive levels of the quadtree. Another approach that has been explored in recent years is the use of artificial neural networks (ANNs). For this approach, simple convolutional network structures such as AlexNet can be used to predict the division flags [12,13] or to predict those flags for the entire CTU [14]. Also, more advanced network architectures, such as ResNet [11] or DenseNet [12], are used to recursively predict the division flags. The further development of convolutional networks used to predict the entire division matrix for a given CTU is also proposed in the literature [15].

This paper presents the further development of the networks based on ResNet [11] and DenseNet [12] architectures for efficiently predicting the partitioning of the entire CTU in an HEVC encoder. The possible tradeoffs to be made when choosing the network structure are demonstrated. Experimental results for diverse modifications of the network structures are presented, along with a comparison with a fully convolutional reference network. The focus is on All Intra coding, where the encoder compresses each video frame separately without motion compensation prediction. All Intra mode can easily be used to encode both still images and videos, providing low latency in encoding and decoding.

## 2. Materials and Methods

### 2.1. Experimental Setup

**Figure 3:**
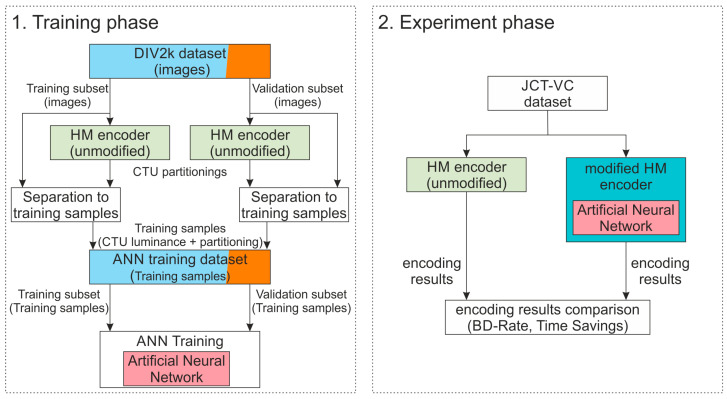
The experiment procedure. Full description is given in the text.

The experiment procedure is presented in Figure 3. In experiments, the modified HEVC encoder [15] is used. This software allows the use of pre-trained ANN models as long as the input and output formats are compatible. Therefore, the input to the proposed ANNs is luminance samples associated with the currently processed CTU block: an image fragment of 64 × 64 pixels. Further, the output is a 4 × 4 × 4 tensor containing the estimated probabilities of a given division depth for the considered block of 64 × 64 pixels constituting a CTU. Such dimensions of the output tensor are determined by the most typical configuration for HEVC. Since the CTU can be divided down to CU blocks of 8 × 8 pixels, the division matrix (Figure 2) can be reduced from size 16 × 16 to 4 × 4. This constitutes the first two dimensions of the output tensor. The size of the last dimension is tied to the maximal depth of the quaternary tree. Since the CTU can be divided down to CU blocks of 8 × 8 pixels, there are four possible division depths for a 64 × 64 pixel CTU (no division at all = 0, division into 32 × 32 pixels blocks = 1, division into 16 × 16 pixels blocks = 2, and division into 8 × 8 pixels blocks = 3).

A number of different networks were prepared based on both ResNet and DenseNet, varying in the details of the structure. A more specific description, with a list of proposed network variations, is presented in Section 2.2.

For training purposes, a dedicated training dataset was prepared, using the DIV2k image dataset [16]. The DIV2k consists of training (800 images) and validation (100 images) subsets. To prepare a dedicated training dataset, the images from the DIV2k dataset were encoded using the HM (version 16.23) [8] in All Intra mode. Then, training samples were composed by pairing a CTU luminance sample (64 × 64 pixels) with a partitioning chosen by the HM. This results in a dedicated training dataset comprising 589,589 samples in the training subset and 66,650 in the validation subset.

It is important to mention that the partitioning for a certain CTU block differs depending on the quantization parameter (QP) value. Therefore, a separate training dataset was prepared for each QP value indicated in the Common Test Conditions for HEVC (CTC) [17]. This means that a separate ANN model is trained for each QP value and then used in evaluation.

All networks were trained for 50 epochs, restarting the optimizer state every five epochs. Adaptive Moment Estimation (ADAM) [18] was used as an optimizer, and the loss function was cross-entropy. The batch size was 64, and training samples were shuffled every epoch. The training was performed using the Tensorflow [19] framework (version 2.5) with GPU acceleration (Python 3.8, CUDA 11.2, cuDNN 8.1, nVidia series 16, 20, and 30). The final model was converted to the corresponding model in LibTorch [20] (version 1.8.1 for CPU).

Once trained, the networks were used to encode the final evaluation with the modified HEVC encoder [15]. The final evaluation was performed with the Joint Collaborative Team on Video Coding (JCT-VC) dataset, defined in CTC [17]. Table 1 presents sequences in JCT-VC. The modified HEVC encoder [15] is based on the HM in version 16.23; thus, this HM version was used to produce reference results. The appropriate CTC for the All Intra scenario was applied without any changes. This means that encoding was performed for four QP values: 22, 27, 32, and 37.

One should mention that a modified HEVC encoder running ANNs uses only CPU, which is limited to a single logical core and a single thread. Such an approach ensures a fair comparison with the HM (single-threaded implementation) encoder [8]. To estimate the performance of the ANN, the results from the modified HM with the tested ANN are compared with the results of the HM for the same sequence and conditions. The following parameters were collected during the final evaluation: the number of additions and multiplications performed in millions of operations, the number of network parameters in thousands of parameters, the changes in the stream produced by the encoder, expressed as a Bjøntegaard delta [21], and finally the encoding time reduction, expressed in the percent of the reference encoder [8] encoding time. The final evaluation was performed on a single platform consisting of an R9 7950X processor with 96 GB of DDR5 RAM and NVME SSD for data storage under the control of Ubuntu in version 24.04.

Since the raw output of the neural network may not be compatible with the HEVC syntax, the output tensor must be adjusted to comply with the HEVC standard requirements. Therefore, during the operation of the modified HEVC encoder, after the ANN processing, it is ensured that the division map is a valid CTU quadtree division. A heuristic algorithm is used to adjust the values. First, the result of the ArgMax function of the output tensor (for the last tensor dimension) is calculated, resulting in a 4 × 4 matrix with division depth values. Then, this matrix is adjusted in the following manner. If the number of division depth values being equal to 0 is greater than 8, then the whole CTU division depth is set to 0. Following, the smaller regions (according to the quaternary tree) are considered with adjusted thresholds for depth values of 1 and 2. At this stage, the matrix is also converted to a 16 × 16 matrix as used internally in the HEVC encoder by multiplicating the values contained in a 4 × 4 matrix.

### 2.2. Examined Network Structures

This research examines two ANN architectures, ResNet [22] and DenseNet [23]. These architectures are more advanced variants of fully convolutional networks, which utilize a shortcut connection or connections between layers in the network. In this research, tested ANNs are composed of specific blocks, according to a given architecture. To ensure the proper size of the output tensor, ANNs are composed of at least four such specific blocks, each paired with a MaxPool layer (pool size and stride both equal 2). Some DenseNet ANNs use only three blocks; thus, the pooling size in the last pooling layer is adjusted accordingly. If not specified in the modification description, only one specific block is applied before the pooling layer. Another restriction is the number of feature maps outputted from the last convolution layer in the ANN, which must be four. Lastly, the last layer in the ANN is always a Softmax. In the proposed network, convolution filters always have a size of 3 × 3, and appropriate padding is applied (a copy of edge samples).

**Figure 4:**
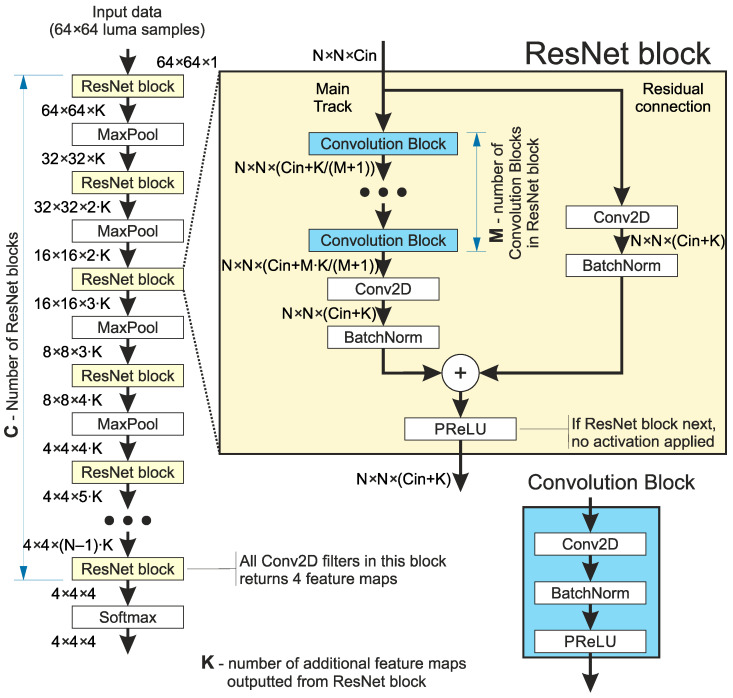
Base architecture of the ResNet network used. Please note that for each “ResNet block” the Conv2D blocks will have a different number of filters to provide the correct number of output layers, as shown on the left.

The first ANN architecture examined in this research is ResNet [24]. A schematic diagram of the ResNet architectures used is presented in Figure 4. The characteristic feature of this architecture is the residual connection. This residual connection conveys feature maps to the end of the block, omitting the main processing track in the block. Within the residual connection, the feature maps can be processed by multiple convolutional layers (or none) [25]. In this research, a residual connection contains a single convolution layer with Batch Normalization. The output of a block is the sum of the feature maps from the main processing track and residual connection, processed by activation (PReLU [26]).

In the description of the ResNet architectures, one will refer to the term Convolution Block as an element of the network. In this paper, this refers to three layers: 2D Convolution, Batch Normalization, and PReLU, connected in the presented order. See the blue inset in Figure 4.

A number of variants were examined in this research. Table 2 presents the list of variants, whose results are presented and discussed in this paper. The number of Convolution Blocks refers to Convolution Blocks used in the main track of the ResNet block.

**Figure 5:**
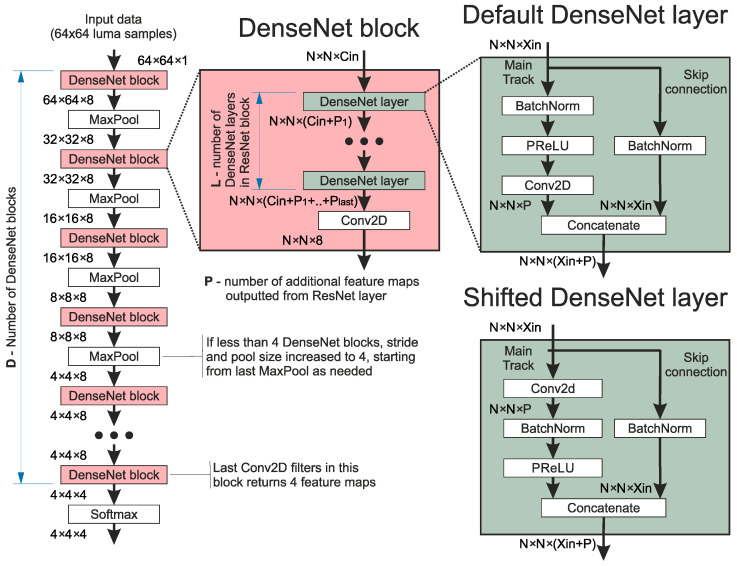
The base architecture of the DenseNet network used. Please note that for “DenseNet layer” and “DenseNet block”, the Conv2D blocks will have a different number of filters, and the MaxPool layer will have a different size to provide the correct number of output layers, as shown on the left.

The second network architecture examined in the research was DenseNet [27]. A schematic diagram of the DenseNet architecture used is presented in Figure 5. The distinctive part of these networks is the dense connection applied between all DenseNet layers with the DenseNet block. This means that consecutive layers in the blocks jointly process all feature maps, both from the input of the block and all previous layers. In this research, the dense connections are achieved by outputting from the layer the concatenation of generated and inputted feature maps of the given layer. The block always ends with a single Conv2D, which processes accumulated feature maps and reduces their number. In this research, the first block always outputs eight feature maps, while the rest output four feature maps.

One should mention that in some architectures, only three DenseNet blocks, paired with pooling layers, are used. Therefore, the pool size and stride are set to four in the last pooling layer to maintain the desired output tensor size.

In the presented research, two types of DenseNet layers were examined. In the first one, referred to as Default, the order of layers in the main track is BatchNorm, PReLU, and Conv2D. In the second one, referred to as Shifted, the order of layers in the main track is Conv2D, BatchNorm, and PReLU. Depending on the type of DenseNet layers used, the number of convolutions in consecutive layers for a given DenseNet block differs. In the case of Default DenseNet layers, the number of convolution filters in consecutive DenseNet layers is 4, 6, 8, 6, 4, 4. When Shifted DenseNet Layers are used, the number of convolution filters in consecutive DenseNet layers is 4, 6, 8, 8, 6, 4. One should mention that when fewer DenseNet layers in the DenseNet block are applied, unnecessary layers are simply removed.

Table 3 presents the list of variants whose results are presented and discussed in this paper.

## 3. Results

All the networks were trained on the DIV2K-based dataset and evaluated in a modified HEVC encoder [15] using the JCT-VC dataset [17], according to the description presented in Section 2.1. None of the sequences from the JCT-VC dataset [17] were used during the training process. In Table 4, we present the results of the experiments as a set of data gathered during the experiment. Please note that the lower the value for the BD-Rate, the better. For Time Saving, the higher the better. In this paper, only Time Savings for encoding are presented, as the decoding time did not change meaningfully.

One should mention that Table 4 includes results for a fully convolutional ANN delivered by the authors of the modified HM encoder [15]. This ANN was evaluated using the exact same procedure as the proposed ANNs. As it is one of the most popular and effective solutions found in the literature and represents a much simpler convolutional architecture, it is an excellent reference point for the ANNs proposed in this paper.

The fully convolutional network [13] used as a reference method has a very similar architecture to the proposed networks. It consists of seven blocks connected in series. The first five are Convolution Blocks (exactly the same as those used in the ResNet architecture), of which the first four are paired with MaxPool. Then, feature maps are split into four equal parts along the vertical and horizontal axes and processed with four independent Convolution Blocks. Further, feature maps are concatenated to restore the previous size and processed with Convolution Blocks but with convolution filters of size 1 × 1. Lastly, Softmax is applied to produce the output tensor.

One should mention that more ANN-based CTU partitioning algorithms are available in the literature. Worth mentioning are the methods presented by Feng [28] (BD-Rate: 1.75%, Time Saving: 60.35%), Xu [29] (BD-Rate: 2.25%, Time Saving: 61.85%), and Wang [30] (BD-Rate: 1.86%, Time Saving: 61.23%). However, certain aspects of the evaluation of results led to not including these results in the comparison. Firstly, models and encoder implementations were not available. The difference in used hardware for evaluation is an important factor that may disrupt the comparison. Secondly, older versions of the HM software used for evaluation (16.20 for Feng [28] and 16.5 for Xu [29] and Wang [30]) differ in partitioning decisions and computing time. Next, for ANN training in the mentioned methods, different training datasets were used. This aspect is crucial, as in this research focus is put strictly on ANN architecture. Lastly, as the scope of this research is on embedded devices, some aspects of ANN in these methods, like number of weights (for Xu [29] and Wang [30]—AlexNet-like architecture) or the number of MAC operations (for Feng [28]), exclude the use of these models in embedded solutions.

**Figure 6:**
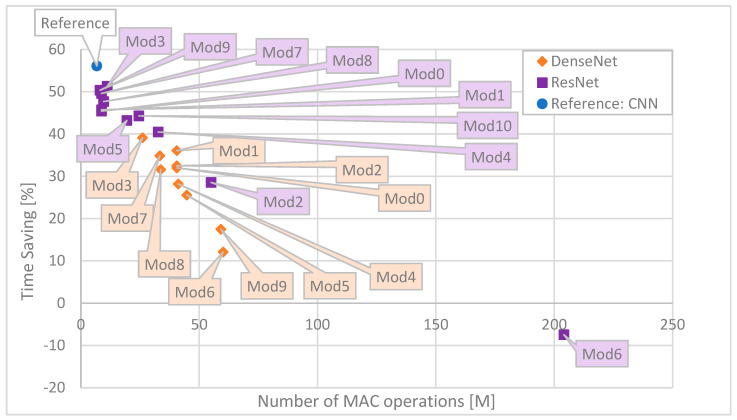
Correspondence between Time Saving and the number of operations performed for each network. The best ones are close to the upper left corner.

**Figure 7:**
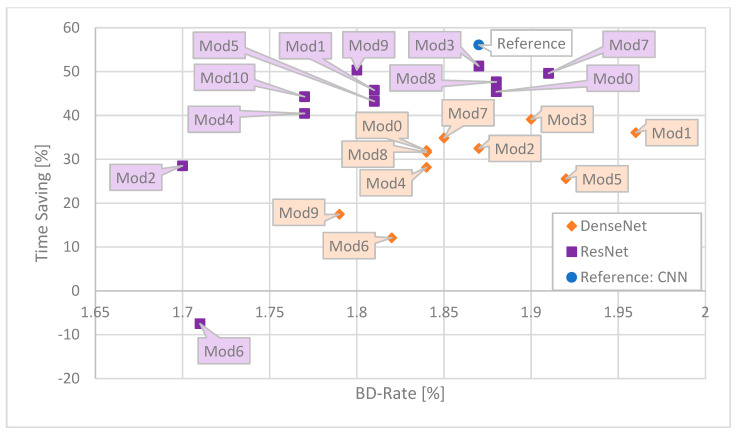
Correspondence between Time Saving and BD-Rate of the resulting bitstream for each network. The best ones are close to the upper left corner.

**Figure 8:**
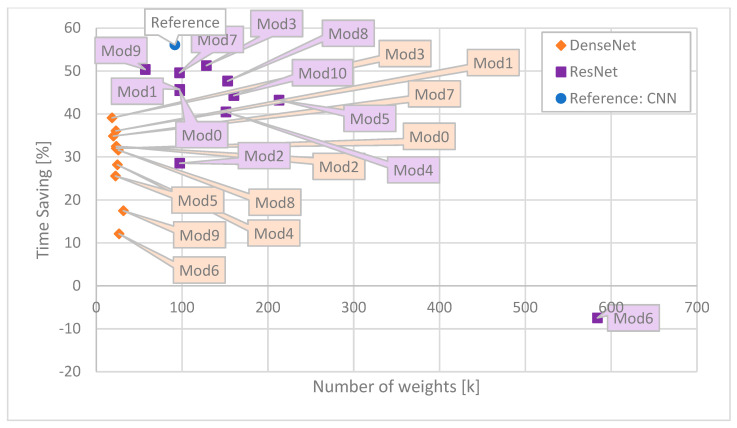
Correspondence between Time Saving and the number of weights for each network. The best ones are close to the upper left corner.

**Figure 9:**
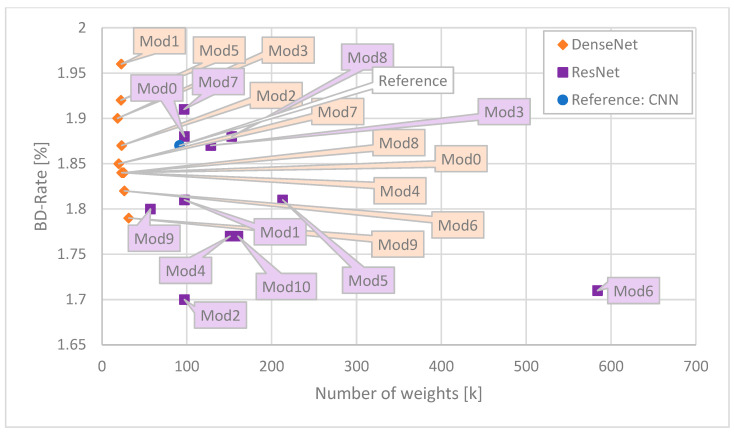
Correspondence between BD-Rate and number of weights for each network. The best ones are close to the lower left corner.

**Figure 10:**
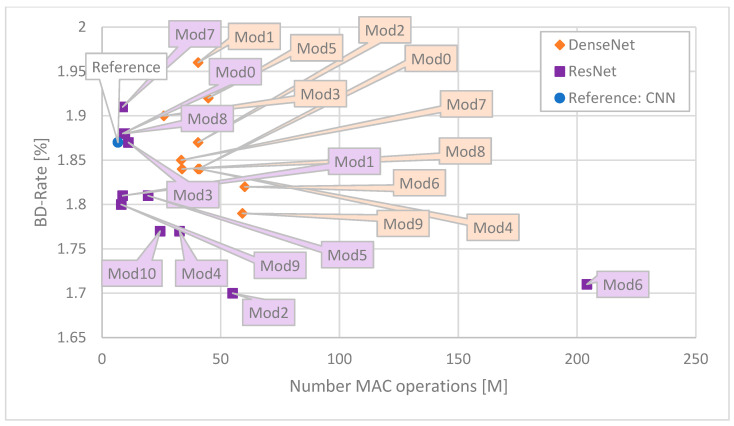
Correspondence between BD-Rate and number of weights for each network. The best ones are close to the lower left corner.

For ease of comparison, the results are presented in the form of graphs. As the most illustrative ones, the following ones are presented: Time Saving as a function of number of MAC operations (Figure 6), Time Saving as a function of BD-Rate (Figure 7), Time Saving as a function of number of weights (Figure 8), BD-Rate as a function of number of weights (Figure 9), and BD-Rate as a function of number of MAC operations (Figure 10).

Regardless of the proposed variant of the ResNet or DenseNet, it can be seen (Figure 6) that the full convolutional network provides the best Time Saving with the fewest number of operations. The ResNet architecture in most variants requires about 30% more MAC operations; however, the Time Savings are smaller by 5 to 10 p.p. compared to a full convolutional network. Despite a very similar number of MAC operations, the results of Time Savings for ResNet Mod0 and Mod9 vary by 5 p.p. The major difference between those two variants is the number of ResNet blocks—Mod9 has one less. On the other hand, the results for Mod10 are very similar to Mod0 (1 p.p. difference) despite a much bigger number of MAC operations. This means that the number of layers is much more impactful on ANN computing time than the number of convolution filters. In our experiments, DenseNet architectures are significantly slower than ResNet-based networks. Despite fewer MAC operations in DenseNet Mod3, the ResNet Mod 4 provides better Time Saving. This means that a greater number of skip connections caused a significant latency in computations, thus deteriorating the Time Savings results. Therefore, the number of residual/dense blocks impacts the Time Savings differently. Considering ResNet, the most complex modification is increasing the number of blocks before the pooling layers. As more blocks process feature maps of bigger dimensions, both the encoding time and number of MAC operations rapidly increase. Having the DenseNet, each additional block significantly increases the number of MACs, which results in ~5 p.p. lower Time Savings per additional block.

A broader perspective on the performance of proposed networks is shown when BD-Rate is considered. As demonstrated in Figure 7, the bitstream produced by the encoder is slightly bigger than the unmodified HM encoder, despite the used network. The two most complex (number of MAC operations) variants of ResNet, Mod 2 and Mod 6, obtained the best results, around 1.7%, which outperforms the reference CNN by ~0.2 p.p. However, the computational complexity of the network is not directly related to the bitstream produced by the encoder. Among the least complex ResNet architectures, in terms of BD-Rate, some of them perform better (e.g., Mod9) and some worse (e.g., Mod 7) compared to the reference CNN architecture. Considering DenseNet architectures, most of the proposed networks outperform the reference CNN, with results comparable to most ResNet architectures. Among the two tested DenseLayer types, the Shifted is more effective. However, ResNet (purple) Mod9 can outperform all but one (orange Mod9) DenseNet network in terms of BD-Rate.

One should recall that both ResNet and DenseNet were tested with AveragePooling (Mod1 for ResNet and Mod 2 for DenseNet) and ReLU as activation (Mod 7 for ResNet and Mod 1 for DenseNet). These modifications did not change the complexity (number of MAC operations) or the size of the networks (number of parameters), as presented in Table 4. Considering Time Saving and BD-Rate, almost none of these modifications improved the performance of the network, as the results are worse compared to the non-modified versions of networks (Mod0) that use PReLU and MaxPool. The exception is ResNet with AveragePooling (Mod1), where BD-Rate is slightly better.

Yet another perspective is given when the number of weights is considered. As shown in Figure 8, the main advantage of the DenseNet architectures is the number of weights. The smallest ResNet architecture (Mod9) is slightly less than twice as big as the biggest DenseNet (Mod9). Moreover, the DenseNet (Mod9) has three times fewer weights than the reference CNN. Therefore, small size does not come with the encoding time, as most ResNet architectures provide higher Time Savings. In some modifications of ResNet, consecutive ResNet blocks produced more feature maps (Mod3, Mod4, and Mod10) as compensation for a lower number of ResNet blocks. One can observe that for such modifications, despite a significant increase in the number of weights, the Time Savings did not change compared to the Default ResNet (Mod0).

Unfortunately, the smallest network (DenseNet—orange—Mod3) causes the encoder to produce a bitstream larger than most of the competitors, as demonstrated in Figure 9. It is worth noting, though, that for hardware-constrained applications, the Time Saving provided by the DenseNet Mod3 network, accompanied by its smallest size, might be more important than the fact that the encoder produces a slightly larger bitstream. Overall, for DenseNet, the bigger the network, the better the BD-Rate. Different observations are made for the ResNet architectures. The BD-Rate result is more related to the block and later setup (Mod2, Mod 4 and Mod10) rather than overall network size (Mod 5 and Mod 6).

When the more important factor is the number of operations and the size of the network (number of weights) is less of an issue, ResNet-based networks will be a better choice (like purple Mod9). This is evidenced in Figure 10. DenseNet architectures provide slightly higher Time Saving with less than half of the number of operations than the best DenseNet (orange Mod3), causing a smaller output bitstream increase. DenseNet Mod3 provides the most Time Saving and is the smallest network among all the DenseNet-based networks in this study, requiring the least MAC operations to compute. On the other hand, its shortcoming is the production of a larger bitstream than most of the other networks in this study.

## 4. Conclusions

Artificial neural networks provide a feasible method of speeding up the compression process of an HEVC coder. In the paper, we presented a comparison of the performance of different artificial neural networks used to predict the divisions in the CTU block for an HEVC encoder in All Intra mode. The results obtained during the presented study show that there is a noticeable difference between the network architectures, both in terms of their performance, size, and complexity in the said application. The studied HEVC All Intra compression mode is especially useful in applications where high-quality images are compressed. The HEVC coder proves to be an effective tool for still image compression. Reducing the complexity of the encoding process through an efficient network predicting the CTU divisions is an important improvement to the encoding process.

However, for some applications, it is important to keep the size of the network as small as practically possible. Although the most obvious method to reduce the size of the whole network is to reduce the number of blocks in the network, our study proves that it is more beneficial to change the overall structure of the network. Although ResNet networks provide better performance than DenseNet networks, trying to reduce the size of the ResNet network will decrease its performance to a point where it is better to change the structure of the network to a slightly worse-performing DenseNet structure that provides a significant reduction in size, time complexity, and number of MAC operations. This is especially important for applications with strict memory and power constraints, like in sensor networks or similar applications.

In future work, we intend to extend the study to the performance improvement of interframe coding in HEVC as well as study the performance of the networks described in this paper in other available encoders.

## Figures and Tables

**Table 1:** The list of the sequences used for evaluation.

JCT-VC Class	Sequence Name	Resolution	Number of Frames	Framerate	Bit Depth
A	NebutaFestival	2560 × 1600	300	60	10
	PeopleOnStreet	2560 × 1600	150	30	8
	SteamLocomotiveTrain	2560 × 1600	300	60	10
	Traffic	2560 × 1600	150	30	8
B	BQTerrace	1920 × 1080	600	60	8
	BasketballDrive	1920 × 1080	500	50	8
	Cactus	1920 × 1080	500	50	8
	Kimono1	1920 × 1080	240	24	8
	ParkScene	1920 × 1080	240	24	8
C	BasketballDrill	832 × 480	500	50	8
	BQMall	832 × 480	600	60	8
	PartyScene	832 × 480	500	50	8
	RaceHorses	832 × 480	300	30	8
D	BasketballPass	416 × 240	500	50	8
	BlowingBubbles	416 × 240	500	50	8
	BQSquare	416 × 240	600	60	8
	RaceHorsesLow	416 × 240	300	30	8
E	FourPeople	1280 × 720	600	60	8
	Johnny	1280 × 720	600	60	8
	KristenAndSara	1280 × 720	600	60	8
	FourPeople	1280 × 720	600	60	8

**Table 2:** Description of the examined architectures of ResNet-based networks. The number of Convolution Blocks refers to the Convolution Block in the main track of the ResNet block.

Network Name	Description	Number of ResNet Blocks (C)	Convolution Blocks in ResNet Block (M)	Number of Additional Feature Maps from ResNet Block (K)
Mod0	Base architecture	7	2	6
Mod1	AvgPool2D as a pooling layer	7	2	6
Mod2	Two ResNet blocks before each MaxPool	8	2	6
Mod3	Single Convolution Block in the ResNet block, more feature maps added by the ResNet block	7	1	8
Mod4	Four Convolution Blocks in the ResNet block, two less ResNet blocks, and more feature maps added by the ResNet block	5	4	10
Mod5	Three Convolution Blocks in the ResNet block, more feature maps added by the ResNet block	7	3	6
Mod6	Three ResNet blocks before MaxPool	12	2	6
Mod7	ReLU as activation	7	2	6
Mod8	Additional ResNet block	8	2	6
Mod9	One ResNet block less	6	2	6
Mod10	More feature maps added by the ResNet block	7	2	12

**Table 3:** Description of the examined architectures of DenseNet-based networks.

Network Name	Description	Number of DenseNet Blocks (D)	Number of DenseNet Layers in Each DenseNet Block (L)	Applied Dense Layer Type
Mod0	Base architecture	3	6	Default
Mod1	ReLU as activation	3	6	Default
Mod2	AvgPool2D as a pooling layer	3	6	Default
Mod3	4 DenseNet blocks, with 4 DenseNet layers in each	4	4	Default
Mod4	Using Shifted DenseNet layers	3	6	Shifted
Mod5	Using 5 DenseNet blocks with 4 DenseNet layers each	5	4	Default
Mod6	Using 6 DenseNet blocks with 4 DenseNet layers each	6	4	Default
Mod7	Using 3 DenseNet blocks with 5 Shifted DenseNet layers each	3	5	Shifted
Mod8	Using 4 DenseNet blocks with 5 Shifted DenseNet layers each	4	5	Shifted
Mod9	Using 5 DenseNet blocks with 5 Shifted DenseNet layers each	5	5	Shifted

**Table 4:** The results of the experiment.

Network	BD-Rate [21] [%]	Time Saving Compared to Original HM [8] [%]	Number of Additions and Multiplications (MAC) Operations (in Millions)	Number of Model Parameters (Weights) (in Thousands)
Full CNN [13]	1.87	56.08	6.76	91.600
ResNet Mod0	1.88	45.41	8.74	97.202
ResNet Mod1	1.81	45.74	8.74	97.202
ResNet Mod2	1.70	28.54	55.00	97.148
ResNet Mod3	1.87	51.27	11.13	128.188
ResNet Mod4	1.77	40.47	32.66	150.992
ResNet Mod5	1.81	43.22	19.44	212.878
ResNet Mod6	1.71	−7.46	204.08	583.968
ResNet Mod7	1.91	49.59	8.74	96.852
ResNet Mod8	1.88	47.67	9.62	152.960
ResNet Mod9	1.80	50.36	8.10	57.086
ResNet Mod10	1.77	44.28	24.49	160.262
DenseNet Mod0	1.84	32.00	40.49	23.170
DenseNet Mod1	1.96	36.08	40.49	22.820
DenseNet Mod2	1.87	32.48	40.49	23.170
DenseNet Mod3	1.90	39.11	26.06	18.330
DenseNet Mod4	1.84	28.19	41.16	24.540
DenseNet Mod5	1.92	25.57	44.75	22.380
DenseNet Mod6	1.82	12.11	60.11	26.420
DenseNet Mod7	1.85	34.86	33.38	19.890
DenseNet Mod8	1.84	31.61	33.73	25.670
DenseNet Mod9	1.79	17.49	59.15	31.450

## Data Availability

Dataset available on request from the authors.

## Abbreviations

The following abbreviations are used in this manuscript:

ADAM	Adaptive Moment Estimation optimizer
AVC	Advanced Video Coding
CPU	Central Processing Unit
CTU	Coding Tree Unit
CU	Coding Unit
GPU	Graphics Processing Unit
HEVC	High Efficiency Video Coding
HM	HEVC Test Model (reference implementation of an HEVC)
JCT-VC	Joint Collaborative Team on Video Coding
QP	Quantization Parameter
VVC	Versatile Video Coding
